# Association Between Age and Complications After Outpatient Colonoscopy

**DOI:** 10.1001/jamanetworkopen.2020.8958

**Published:** 2020-06-25

**Authors:** Natalia Causada-Calo, Kirles Bishay, Siwar Albashir, Ahmed Al Mazroui, David Armstrong

**Affiliations:** 1 Division of Gastroenterology, St Michael’s Hospital, University of Toronto, Toronto, Ontario, Canada; 2 Division of Gastroenterology, Department of Medicine, McMaster University, Hamilton, Ontario, Canada; 3 Farncombe Family Digestive Research Institute, Department of Medicine, McMaster University, Hamilton, Ontario, Canada

## Abstract

**Importance:**

There are insufficient data describing the incidence and risk factors of postcolonoscopy complications in older individuals.

**Objective:**

To assess the association between older age (≥75 years) and the risk of postcolonoscopy complications.

**Design, Setting, and Participants:**

This population-based retrospective cohort study included adults (≥50 years) undergoing outpatient colonoscopy between April 2008 and September 2017, identified from Ontario administrative databases. Individuals with inflammatory bowel disease and hereditary colorectal cancer syndromes were excluded. The study population was subdivided into a colorectal cancer screening–eligible cohort (patients aged 50-74 years) and an older cohort (patients aged ≥75 years). The statistical analysis was conducted from December 2018 to September 2019.

**Exposures:**

Older age (≥75 years).

**Main Outcomes and Measures:**

The primary outcome was postcolonoscopy complications, defined as the composite of hospitalization or emergency department visits in the 30-day period after the outpatient colonoscopy. Secondary outcomes included incidence of surgically treated colorectal cancer and all-cause mortality at 30 days. Independent variables associated with postcolonoscopy complications were also assessed.

**Results:**

The study sample included 38 069 patients; the mean (SD) age was 65.2 (10.1) years, there were 19 037 women (50.0%), and 27 831 patients (73.1%) underwent a first colonoscopy. The cumulative incidence of complications was 3.4% (1310 patients) in the overall population, and it was higher in individuals aged 75 years or older (515 of 7627 patients [6.8%]) than in screening-eligible cohort (795 of 30 443 patients [2.6%]) (*P* < .001). Independent risk factors for postcolonoscopy complications were age 75 years or older (odds ratio [OR], 2.3; 95% CI, 2.0-2.6), anemia (OR, 1.4; 95% CI, 1.2-1.7), cardiac arrhythmia (OR, 1.7; 95% CI, 1.2-2.2), congestive heart failure (OR, 3.4; 95% CI, 2.5-4.6), hypertension (OR, 1.2; 95% CI, 1.0-1.5), chronic kidney disease (OR, 1.8; 95% CI, 1.1-3.0), liver disease (OR, 4.7; 95% CI, 3.5-6.5), smoking history (OR, 3.2; 95% CI, 2.4-4.3), and obesity (OR, 2.3; 95% CI, 1.2-4.2). The number of previous colonoscopies was associated with a lower risk of complications (OR, 0.9; 95% CI, 0.7-1.0). The incidence of surgically treated colorectal cancer was higher in the older cohort than the screening-eligible cohort (119 patients [1.6%] vs 144 patients [0.5%]; *P* < .001). All-cause mortality rates were 0.1% overall (39 patients) and 0.1% (19 patients) for individuals aged 50 to 74 years and 0.2% (20 patients) for those aged 75 years and older (*P* < .001).

**Conclusions and Relevance:**

In this population-based cohort study of individuals living in southern Ontario, age of 75 years and older was associated with a higher risk of 30-day postprocedure complications after outpatient colonoscopy. These findings suggest that the decision to perform a colonoscopy should be carefully considered in patients older than 75 years, especially in the presence of comorbidities. Further studies are needed to better understand the benefits of invasive procedures as opposed to less invasive approaches for colorectal cancer screening and surveillance among older patients.

## Key Points

**Question:**

Are individuals aged 75 years and older at a higher risk of 30-day postcolonoscopy complications compared with those aged 50 to 74 years?

**Findings:**

In a cohort study of 38 069 patients who underwent outpatient colonoscopy between 2008 and 2017, the cumulative incidence of postcolonoscopy complications at 30 days was higher in individuals aged 75 years and older compared with those aged 50 to 74 years. The odds of postcolonoscopy complications were also significant for individuals with baseline comorbidities compared with those without comorbidities.

**Meaning:**

The findings of this study suggest that the decision to perform colonoscopy should be considered carefully for older patients, particularly in the presence of comorbidities, given the higher risk of postcolonoscopy complications.

## Teaser

This cohort study examines the association between older age (≥75 years) and the risk of postcolonoscopy complications in a sample of outpatients in Ontario, Canada.

## Introduction

Colorectal cancer is the third most common cancer diagnosed in men and women, and the second leading cause of cancer-related deaths in North America, with 53 200 deaths expected to occur during 2020.^1^ Because of the considerable burden of colorectal cancer, there has been widespread uptake of screening programs, with several studies showing mortality benefit from fecal testing, sigmoidoscopy, or colonoscopy.^2,3,4,5,6,7,8,9,10,11^ Most society guidelines recommend colorectal cancer screening starting at age 50 years through 75 years for individuals with average risk.^12,13,14^ Postpolypectomy surveillance colonoscopy is performed as a result of an estimated 10% recurrence rate per year of adenomatous polyps to detect and remove these polyps and to diagnose metachronous or interval colorectal cancers.^15,16,17,18^ Despite many years of colonoscopy use, there is a paucity of data on surveillance colonoscopy and its complications among older patients.^19,20,21^

Overall, the effectiveness of colorectal cancer–related colonoscopy is based primarily on an individual’s life expectancy and risk of colorectal cancer.^22^ Patients aged 50 to 59 years are perceived to have a lower absolute benefit from screening because of the lower incidence of colorectal cancer in this age group compared with that in older patients. The most recent Canadian guidelines^13^ make a weak recommendation to screen individuals aged 50 to 59 years compared with a strong recommendation for screening those aged 60 to 75 years, whereas a weak recommendation is made to not screen after age 75 years. On the other hand, the older population is heterogeneous in functional status and burden of comorbidities, and thus, age alone cannot be the sole criterion for decision-making. Clinicians then face the challenge of selecting patients who would benefit from a colonoscopy despite limited information from current guidelines.

The main objective of this study was to describe the incidence and independent risk factors of postcolonoscopy complications among adult patients undergoing colonoscopy in the outpatient setting, using a large administrative population-based data set. Current recommendations for colorectal cancer screening propose an age cutoff of 75 years to either stop or individualize screening, given the unclear benefit of screening and surveillance after this age.^12,13,14^ Therefore, we considered age 75 years or older as a key variable in our analyses. As a secondary objective, we sought to describe the risk-benefit ratio by contrasting the incidence of complications and surgically treated colorectal cancers. We hope that our findings will help identify which patients are more likely to develop postcolonoscopy complications. Ultimately, this information could contribute to understanding the balance between the risks and benefits of outpatient colonoscopy among older patients.

## Methods

### Study Design, Setting, and Participants

We conducted a population-based retrospective cohort study of individuals who underwent colonoscopy between April 2008 and September 2017. We included all consecutive adults (≥50 years) who underwent outpatient first-time or surveillance colonoscopies in the area of Hamilton, Ontario, Canada. Data were obtained from the following databases: Ontario Health Insurance Plan, Registered Persons Database, the Canadian Institute for Health Information Discharge Abstract Database, and the Canadian National Ambulatory Care Reporting System. The Registered Persons Database records age, sex, postal code information, and vital statistics for Ontario residents with a valid Ontario Health Insurance Plan number. The Canadian Institute for Health Information Discharge Abstract Database records data on hospital discharges (including deaths, sign-outs, and transfers) and diagnostic codes based on the *International Classification of Diseases, 10th Revision, Clinical Modification*. The National Ambulatory Care Reporting System database contains data for all hospital- and community-based ambulatory care, including same-day surgery, outpatient and community-based clinics, and emergency departments. A detailed explanation of the databases and codes is provided in eTable 1 in the Supplement. The strategy we used to select patients undergoing outpatient colonoscopy, in the absence of recorded relevant symptoms, was based on previously validated algorithms.^23,24^ In the case of patients who underwent multiple surveillance colonoscopies, the last surveillance procedure was considered for the analysis of the end points of interest, and the total number of colonoscopies was recorded as a patient-level covariate. Once the cohort was assembled, we divided it into 2 different subcohorts according to age categories as follows: aged 50 to 74 years (colorectal cancer screening–eligible cohort) and aged 75 years and older (older cohort). Individuals with a history of inflammatory bowel disease or a hereditary colorectal cancer syndrome were excluded because they would not be representative of the average risk population. To minimize loss to follow-up and missing data on postcolonoscopy complications, we captured data on all hospital admissions and emergency department visits in a broader area in Ontario, including Hamilton, Niagara, Haldimand, Brant, Burlington, and most of Norfolk, which represents a population of 1.5 million. This study was conducted according to the Strengthening the Reporting of Observational Studies in Epidemiology (STROBE) reporting guideline and was approved by the Hamilton Integrated Research Ethics Board, including a waiver for patient consent because all personally identifiable information was removed from the data sets.

### Study Variables and Measurements

We collected baseline demographic and clinical characteristics recorded at the first colonoscopy or at the last surveillance colonoscopy for individuals who had more than 1 colonoscopy performed during the study period. The primary outcome for this study was the cumulative incidence of postcolonoscopy complications, defined as the composite of unplanned hospital admissions or emergency department visits at 30 days after outpatient colonoscopy. We excluded from this definition planned admissions related to the surgical treatment of colorectal cancer. Furthermore, we sought to identify independent factors associated with 30-day postcolonoscopy complications. A priori potential factors were age (dichotomized as 50-74 years vs ≥75 years), sex (women vs men), and baseline comorbidities such as coronary artery disease, anemia, liver disease, obesity, congestive heart failure, chronic kidney disease, cardiac arrhythmia, hypertension, and smoking history (present or past). The secondary outcome was the cumulative incidence of surgically treated colorectal cancer at 30 days in both cohorts. Also, we sought to identify the main reasons for postcolonoscopy complications, including principal diagnoses of gastrointestinal tract hemorrhage, cardiovascular complications, sepsis, and bowel perforation, none of which were related to a diagnosis of colorectal cancer (eTable 1 in the Supplement). Finally, we defined mortality as all-cause mortality at 30 days.

### Statistical Analysis

Continuous variables, such as age and number of previous colonoscopies, are presented as means and SDs and categorical data are presented as proportions. Patients’ baseline characteristics were compared with standardized mean differences. For hypothesis testing, we used the χ^2^ and Fisher exact tests for categorical variables, and *t* test or the Wilcoxon rank sum test for continuous variables, as appropriate.

A multivariable logistic regression model was built to identify independent factors associated with postcolonoscopy complications. The criteria to introduce variables into the model were based primarily on clinical relevance and previous literature, important baseline epidemiologic variables (age and sex), and a significant association in the univariable analysis (at a significance level of *P* = .20). We included all variables meeting these criteria into a first logistic regression model (custom model) and subsequently eliminated, 1 at a time, the variables that were not statistically significant in the model (Wald test; significance level *P* < .05). Finally, we reached a point at which all the variables in the model were statistically significant (main effect model). We assessed for multicollinearity between the factors by estimating the variance inflation factor. The calibration (or goodness of fit) of the model was evaluated with the Hosmer-Lemeshow test. As an alternative way to deal with potential baseline confounding of comorbidity, we performed a secondary analysis by matching each exposed patient (≥75 years) with a nonexposed patient(<75 years) with respect to the number of comorbidities (eAppendix in the Supplement).

Given that current evidence does not support a clear age cutoff at which the risks of colonoscopy increase significantly, we explored the relationship between age and postcolonoscopy complications with 2 different approaches. First, we treated age as a continuous variable and performed an exploratory analysis using locally weighted scatterplot smoothing by computing and plotting smoothed points; this is a nonparametric method that fits multiple regressions in local neighborhoods and provides a smooth curve that displays the estimated probability of postcolonoscopy complication.^25^ The main objective of this analysis was to visually explore the graph to decide whether a clear age cutoff, determined by a change in the slope of the curve, could be identified. Second, we grouped individuals by age into 5-year intervals and estimated the incidence of postcolonoscopy complications in each age interval. We estimated the cumulative incidence of all-cause mortality at 30 days; patients who underwent surgery for colorectal cancer were censored for this estimation because the surgical intervention could increase the risk of mortality independently of any colonoscopy-related risk.

All statistical tests were 2 sided, and *P* < .05 was considered significant. All analyses were conducted with Stata version 13 (StataCorp) and were conducted from December 2018 to September 2019.

## Results

### Description of the Study Population

**Figure 1.**
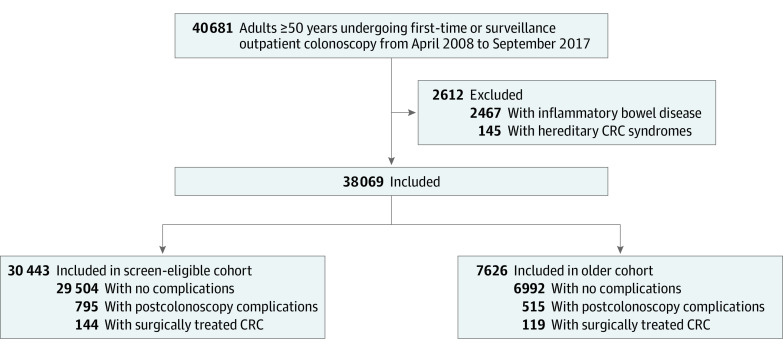
Selection of Study Population CRC indicates colorectal cancer.

We identified 40 681 patients aged 50 years or older who had undergone outpatient colonoscopy between April 2008 and September 2017; 2467 patients (6.1%) were excluded for a previous diagnosis of inflammatory bowel disease and 145 (0.4%) for a hereditary colorectal cancer syndrome (Figure 1). Hence, 38 069 patients were included in the analysis. The mean (SD) age was 65.2 (10.1) years, 19 037 (50.0%) were women, and 27 831 (73.1%) underwent a first colonoscopy (Table 1). On division into the 2 subcohorts, the colorectal cancer screening–eligible cohort comprised 30 443 patients (74.8%; 15 149 [49.7%] women) with a mean (SD) age of 61.4 (7.0) years, and the older cohort comprised 7626 patients (25.2%; 3888 [50.9%] women) with a mean (SD) age of 80.5 (4.3) years. The proportion of patients undergoing first-time colonoscopies was significantly higher in the colorectal cancer screening–eligible cohort than in the older cohort (22 556 [74.1%] vs 5265 [69.0%]; *P* < .001).

**Table 1.** Baseline Characteristics

Characteristic	No. (%)			SMD
	Total (N = 38 069)	CRC screening-eligible cohort (n = 30 443)	Older cohort (n = 7626)	
Age, y				
Mean (SD)	65.2 (10.1)	61.4 (7.0)	80.5 (4.3)	3.26
50-54	6381 (16.8)	6381 (21.0)	NA	NA
55-59	6585 (17.3)	6585 (21.6)	NA	NA
60-64	6482 (17.0)	6482 (21.3)	NA	NA
65-69	5887 (15.5)	5887 (19.3)	NA	NA
70-74	5108 (13.4)	5108 (16.8)	NA	NA
75-79	3752 (9.9)	NA	3752 (49.2)	NA
80-84	2418 (6.4)	NA	2418 (31.7)	NA
85-89	1176 (3.1)	NA	1176 (15.4)	NA
≥90	280 (0.7)	NA	280 (3.7)	NA
Women	19 037 (50.0)	15 149 (49.8)	3888 (51.0)	0.024
First-time colonoscopy	27 831 (73.1)	22 566 (74.1)	5265 (69.0)	−0.112
Previous colonoscopies, mean (SD), No.	1.3 (0.6)	1.2 (0.4)	1.3 (0.7)	0.094
Anemia	2306 (6.1)	1382 (4.5)	924 (12.1)	0.276
Hypertension	2170 (5.7)	1475 (4.8)	695 (9.1)	0.168
Heart failure	311 (0.8)	130 (0.4)	187 (2.5)	0.166
Cardiac arrhythmia	450 (1.2)	153 (0.5)	297 (3.9)	0.232
CAD	258 (0.7)	115 (0.4)	143 (1.9)	0.142
Smoking history	366 (0.1)	169 (0.6)	197 (2.6)	0.163
COPD	129 (0.3)	55 (0.2)	74 (1.0)	0.104
Liver disease	352 (0.9)	315 (1.0)	37 (0.5)	−0.063
CKD	107 (0.3)	44 (0.1)	63 (0.8)	0.0981
Obesity	105 (0.3)	95 (0.3)	10 (0.1)	−0.0384
Family history of CRC or polyps	7564 (19.9)	7052 (23.2)	512 (6.7)	−0.474

Abbreviations: CAD, coronary artery disease; CKD, chronic kidney disease; COPD, chronic obstructive pulmonary disease; CRC, colorectal cancer; NA, not applicable; SMD, standardized mean difference.

There were significant differences in the distribution of comorbidities in the 2 subcohorts. Patients in the older cohort had a higher burden of baseline comorbidities than patients in the colorectal cancer screening–eligible cohort (eg, anemia: 924 [12.1%] vs 1382 [4.5%]; hypertension: 695 [9.1%] vs 1475 [4.8%]; cardiac arrhythmia: 297 [3.9%] vs 153 [0.5%]), with the exception of liver disease and obesity, which were more common in the colorectal cancer screening–eligible cohort than in the older cohort (liver disease: 315 [1.0%] vs 37 [0.5%]; obesity: 95 [0.3%] vs 10 [0.1%]) (Table 1).

### Risk of Postcolonoscopy Complications at 30 Days

A total of 1310 patients (3.4%) experienced a postcolonoscopy complication during the 30 days after outpatient colonoscopy. The cumulative incidence of complications was significantly higher in patients aged 75 years or older (515 of 7627 [6.8%] vs 795 of 30 443 [2.6%]; *P* < .001). The most common complications are detailed in Table 2. The overall incidence of postcolonoscopy bleeding was low (141 patients [0.4%]), but it was significantly higher in the older cohort than in the colorectal cancer screening–eligible cohort (65 [0.9%] vs 76 [0.3%]; *P* < .001). Likewise, the overall incidence of bowel perforation was low (18 patients [0.05%]); however, in this case there was no significant difference between the older and colorectal cancer screening–eligible cohort (6 [0.08%] vs 12 [0.04%]; *P* = .20). Finally, the incidence of cardiovascular complications was 0.7% (274 patients), and it was significantly higher in the older cohort than in the colorectal cancer screening–eligible cohort (136 [1.8%] vs 138 [0.5%]; *P* < .001). Of the 274 patients admitted for cardiovascular-related complications, the most common diagnoses were heart failure (90 patients [32.8%]), myocardial infarction (54 patients [19.7%]), and thromboembolic disease (50 patients [18.2%]) (eTable 2 in the Supplement).

**Table 2.** Description of 30-Day Postcolonoscopy Complications

Characteristic	No. (%)			*P* value^a^
	Total (N = 38 069)	CRC screening-eligible cohort (n = 30 443)	Older cohort (n = 7626)	
Admission or ED visit	1310 (3.4)	795 (2.6)	515 (6.8)	<.001
Postcolonoscopy bleeding	141 (0.4)	76 (0.2)	65 (0.9)	<.001
Bowel perforation	18 (0.05)	12 (0.04)	6 (0.08)	.15
Cardiovascular-related admissions	274 (0.7)	138 (0.5)	136 (1.8)	<.001
Nongastrointestinal malignancy	149 (0.4)	97 (0.3)	52 (0.7)	<.001
Infection-related admissions	148 (0.4)	93 (0.3)	95 (1.2)	<.001
Colorectal surgery^b^	65 (0.2)	47 (0.2)	18 (0.2)	.12
Nonsurgically treated CRC	20 (0.1)	13 (0.04)	7 (0.1)	.09
Diverticular disease	27 (0.07)	21 (0.07)	6 (0.08)	.78
Palliative care	27 (0.07)	19 (0.06)	8 (0.1)	.21
Other	401 (1.1)	279 (0.9)	122 (1.6)	<.001

Abbreviations: CRC, colorectal cancer; ED, emergency department.

^a^ Hypothesis testing was carried out with χ^2^ or Fisher exact test.

^b^ Unrelated to CRC.

We compared the risk of postcolonoscopy complications between patients who underwent first-time and surveillance colonoscopies. The risk was higher after a first colonoscopy in both the full cohort (990 of 27 831 [3.6%] vs 320 of 10 238 [3.1%]; *P* = .04) and in the older cohort (395 of 5262 [7.5%] vs 120 of 2361 [5.1%]; *P* < .001); however, in the colorectal cancer screening–eligible group, the risk of postcolonoscopy complication was similar for first and surveillance colonoscopies (594 of 22 566 [2.6%] vs 200 of 7877 [2.5%]; *P* = .64).

**Figure 2.**
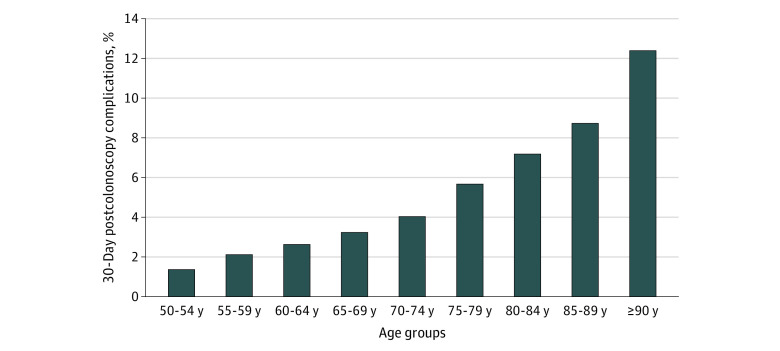
Association of Age With Cumulative Incidence of Postcolonoscopy Complications at 30 Days

To explore the association between age and the probability of postcolonoscopy complications at 30 days in our population, we performed a locally weighted scatterplot (eFigure in the Supplement). We observed that the estimated probability of a complication increased almost linearly with age, and by visual inspection, there appeared to be a mild change in the slope, indicating an increase in the probability of a complication between the ages of 76 and 78 years. This was also evident when we analyzed the association between age, divided into 5-year intervals, and postcolonoscopy complications (Figure 2). The incidence increased from 205 of 5108 patients (4.0%) aged 70 to 74 years to 210 of 3752 patients (5.6%) aged 75 to 79 years and 170 of 2248 patients (7.0%) aged 80 to 84 years.

We also analyzed time to complication after colonoscopy. Overall, 135 patients (10.3%) were admitted or had an emergency department visit on the same day of the colonoscopy, whereas the overall median (interquartile range) time to a complication was 12 (5-21) days, and it was significantly shorter in the older cohort (median [interquartile range] 11 [4-21] days vs 13 [5-22] days; *P* = .008). Last, a total of 39 patients (0.1%) died, and the overall incidence of mortality was significantly higher in the older cohort than the colorectal cancer screening–eligible cohort (17 of 7626 [0.2%] vs 19 of 30 443 [0.1%]; *P* < .001).

### Independent Factors Associated With Postcolonoscopy Complications

We performed a multivariable logistic regression analysis to identify independent factors associated with postcolonoscopy complications (Table 3). The *P* value for the Hosmer-Lemeshow test was .89, indicating that we could not reject the null hypothesis of no difference between estimated and observed. To test the discrimination of the model, we constructed a receiver operating characteristic curve. The area under the curve for the final model was 0.7. In the final model, the overall independent risk factors for postcolonoscopy complications were age 75 years or older (odds ratio [OR] 2.3; 95% CI, 2.0-2.6), anemia (OR, 1.4; 95% CI, 1.2-1.7), cardiac arrhythmia (OR, 1.7; 95% CI, 1.2-2.2), congestive heart failure (OR, 3.4; 95% CI, 2.5-4.6), hypertension (OR, 1.2; 95% CI, 1.0-1.5), chronic kidney disease (OR, 1.8; 95% CI, 1.1-3.0), liver disease (OR, 4.7; 95% CI, 3.5-6.5), smoking history (OR, 3.2; 95% CI, 2.4-4.3), and obesity (OR, 2.3; 95% CI, 1.2-4.2). A greater number of previous colonoscopies was associated with a significantly lower risk of complications (OR, 0.9; 95% CI, 0.7-1.0). Coronary artery disease and smoking history were shown to be collinear; because smoking remained significant in the final model, we included it, rather than coronary artery disease, for the final analysis.

**Table 3.** Independent Factors Associated With Postcolonoscopy Complications at 30 Days

Characteristic	OR (95% CI)		
	Unadjusted	Adjusted	
		Custom model^a^	Final model^b^
Age (≥75 vs 50-74 y)	2.7 (2.4-3.0)	2.3 (2.1-2.6)	2.3 (2.0-2.6)
Women vs men	1.0 (0.9-1.2)	1.1 (0.9-1.2)	NA
First-time vs surveillance colonoscopy	1.1 (1.0-1.3)	1.0 (0.9-1.2)	NA
No. of previous colonoscopies	0.8 (0.7-0.9)	0.9 (0.8-1.0)	0.9 (0.7-1.0)
Anemia^c^	2.4 (2.0-2.9)	1.4 (1.2-1.7)	1.4 (1.2-1.7)
Cardiac arrhythmia^c^	8.9 (7.1-11.1)	2.1 (1.5-2.8)	1.7 (1.2-2.2)
CAD^c^	9.2 (7.0-12.1)	0.9 (0.3-2.1)	NA
Heart failure^c^	13.0 (10.1-16.7)	3.4 (2.5-4.6)	3.4 (2.5-4.6)
Hypertension^c^	2.3 (1.9-2.7)	1.2 (1.0-1.5)	1.2 (1.0-1.5)
CKD^c^	9.8 (6.3-15.2)	1.8 (1.1-3.0)	1.8 (1.1-3.0)
Liver disease^c^	5.5 (4.1-7.3)	4.9 (3.6-6.8)	4.7 (3.5-6.5)
COPD^c^	10.9 (7.3-16.1)	0.8 (0.3-1.8)	NA
Smoking history^c^	10.9 (8.6-13.8)	4.1 (1.6-10.5)	3.2 (2.4-4.3)
Obesity^c^	5.2 (3.0-8.8)	2.4 (1.2-4.5)	2.3 (1.2-4.2)

Abbreviations: CAD, coronary artery disease; CKD, chronic kidney disease; COPD, chronic obstructive pulmonary disease; NA, not applicable; OR, odds ratio.

^a^ Variables included in the custom model were age (≥75 vs 50-74 years), sex (women vs men), type of colonoscopy (first time vs surveillance), number of previous colonoscopies, anemia, cardiac arrhythmia, CAD, heart failure, hypertension, CKD, liver disease, COPD, smoking history, and obesity.

^b^ Variables included in the final model were age (≥75 vs 50-74 years), type of colonoscopy (first time vs surveillance), number of previous colonoscopies, anemia, cardiac arrhythmia, heart failure, hypertension, CKD, liver disease, smoking history, and obesity.

^c^ Binary variable (ie, yes or no) in which the reference group corresponds to the absence of that characteristic.

To assess the potential confounding effect of comorbidity, we performed a secondary analysis by matching exposed and nonexposed patients (age ≥75 vs 50-74 years) on baseline comorbidity. In the matched cohort, the association between older age and postcolonoscopy complications remained significant (OR, 2.9; 95% CI, 2.5-3.5) (eAppendix and eTable 3 in the Supplement)

### Rate of Surgically Treated Colorectal Cancer

Overall, 263 patients (0.7%) underwent surgery for colorectal cancer in the 30 days after colonoscopy. The proportion of patients who had surgery was significantly higher after a first-time colonoscopy compared with surveillance colonoscopy (219 [0.8%] vs 44 [0.4%]; *P* < .001). When the age cohorts were compared, a greater proportion of older patients underwent surgery at 30 days (119 [1.6%] vs 144 [0.5%]; *P* < .001). Furthermore, the incidence was higher after first-time colonoscopy than surveillance colonoscopy in both the colorectal cancer screening–eligible cohort (120 [0.5%] vs 24 [0.3%]; *P* = .01) and the older cohort (99 [1.9%] vs 20 [0.9%]; *P* = .001) (eTable 4 in the Supplement).

## Discussion

This population-based retrospective cohort study compared the cumulative incidence of postcolonoscopy complications, defined as 30-day unplanned hospital admission or emergency department visit after outpatient colonoscopy, between colorectal cancer screening–eligible and older cohorts. Overall, we found that patients aged 75 years and older had higher odds (2.3) of postcolonoscopy complications compared with those aged 50 to 74 years. Independent risk factors for postcolonoscopy complications were the presence of anemia, cardiac arrhythmia, heart failure, hypertension, chronic kidney disease, smoking history, liver disease, and obesity. A greater number of previous colonoscopies was associated with a reduced risk of postcolonoscopy complications, and the cumulative incidence of surgically treated colorectal cancer was considerably lower compared with that of postcolonoscopy complications.

The number and proportion of older adults is increasing worldwide; the older population is projected to double by 2050 and triple by 2100.^26^ Current guidelines provide ambiguous recommendations for both screening and surveillance colonoscopy in individuals older than 75 years, largely because of uncertainty regarding the overall benefits of colonoscopy past this age.^13,14,27,28^ Because chronologic age alone cannot stratify risks reliably^29,30^ and colorectal cancer incidence increases steadily with age, identifying clinical characteristics that aid decision-making regarding the use of colonoscopy among older patients is of the utmost importance for patients and providers.

Our findings are in line with previous research.^31,32^ We found the risk of postcolonoscopy complications to be twice as high in patients aged 75 years or older compared with that of patients aged 50 to 74 years. Given the paucity of randomized clinical trials evaluating the effectiveness of screening colonoscopy in the elderly, García-Albéniz et al^19^ conducted a simulated randomized trial comparing screening colonoscopy to no screening in patients aged 70 to 79 years. In this study, the risk of gastrointestinal and cardiovascular complications was almost twice as high for patients aged 75 to 79 years than for those aged 70 to 74 years. At the same time, the benefit of colorectal cancer diagnosis in the older group was also low compared with the risk of complications. Our findings are similar to those of this study in terms of colorectal cancer diagnosis; however, the incidence of complications in individuals older than 75 years in our study was higher (6.8% vs 1%). A potential explanation for this discrepancy is first how we defined complications at 30 days. We considered all hospital admissions or emergency department visits, whereas García-Albéniz et al^19^ defined complications as admissions or emergency department visits for gastrointestinal or cardiovascular events. Second, we included 3874 individuals aged 80 years or older, who are more likely to experience postcolonoscopy complications than younger patients.^33^ We also analyzed the risk of complications by excluding from this definition admissions unrelated to cardiovascular and gastrointestinal events; the incidence of these complications was 2.4% for patients aged 70 to 79 years, which is closer to the findings by García-Albéniz and colleagues.^19^

We observed that the risk of complications increased steadily with age, particularly in individuals older than 75 years, with a more pronounced increase after the age of 80 years when we analyzed the rate of complications according to age divided in 5-year intervals (and after the ages of 76-78 years when considering age as a continuous variable). In a systematic review that examined postcolonoscopy complications in the elderly,^34^ the rate of composite adverse events (perforation, bleeding, and cardiovascular or pulmonary events) also increased with age. Another large, population-based, matched cohort study^35^ reported 75% higher risk of serious gastrointestinal adverse events for individuals aged 80 to 84 years compared with those aged 66 to 69 years. Overall, despite great heterogeneity across studies and different age group comparisons, these findings indicate that older patients have an elevated risk for an adverse event associated with colonoscopy, and that ages between 75 and 80 years are potential inflexion points for risk considerations.

Last, we observed that cardiovascular comorbidities were independently associated with postcolonoscopy complications and that, in comparison with younger individuals, those older than 75 years had an almost 4-fold increased incidence of cardiovascular complications (0.5% vs 1.8%). These findings are in concordance with the results reported by Sharma and colleagues.^36^ Their study analyzed 174 255 unique colonoscopies and reported that age 60 years or older was associated with an 80% higher risk of postprocedure pulmonary and cardiovascular complications.

Regarding to the risks and benefits pertaining to surveillance colonoscopy in the elderly, the current evidence is much more limited. A large population-based retrospective study^37^ revealed that individuals older than 75 years have a 28% higher risk of postsurveillance colonoscopy hospitalization compared with younger individuals. As did we, the authors concluded that the rate of complications exceeded the rate of colorectal cancer diagnosis in the elderly. In contrast with our findings, they observed a slightly lower hospitalization rate (3.8%) after surveillance colonoscopies. This was perhaps related to the inclusion of a relatively healthier population, given that the patients belonged to a private insurance system.

### Strengths and Limitations

This study has a number of strengths. It was a large population-based study that analyzed the risk-benefit equation of colorectal cancer screening and surveillance colonoscopy in a population of special interest, for which data are limited and current guidelines do not aid decision making.

Our study has several limitations. First, as in any observational study using administrative data, our estimates are prone to confounding; however, our primary objective was not to determine causality, but rather to describe the risk of complications after colonoscopy. In particular, we acknowledge that the burden of comorbidities can confound the association between older age and postcolonoscopy complications. To account for this, in addition to the multivariable logistic regression model, we conducted a secondary analysis matching for number of comorbidities, which demonstrated that the association between older age and risk of postcolonoscopy complications remained significant. We defined postcolonoscopy complications as the composite of all-cause hospitalizations or emergency department visits at 30 days, a definition that we believe to be well documented in all administrative databases and that has been used by several studies in the field.^19,37^ We were unable to obtain a more detailed description of the admission diagnoses and baseline comorbidities, and some could have been underreported. However, we do not think this represents a systematic error. Unfortunately, detailed characterization of the symptoms that may have prompted colonoscopy in some patients (ie, changes in bowel habits) is limited in administrative databases, and if these symptoms were distributed unequally between groups, it could be considered a confounding variable for the association between age and postcolonoscopy complications. To limit this possibility, the administrative codes we used to select our cohort were based on previously validated strategies to exclude patients who underwent colonoscopies because of symptoms.^23,24^

Second, we were unable to capture all the patients who received a diagnosis of different stages of colorectal cancer; our report is limited to those who underwent surgery within 30 days after colonoscopic diagnosis, and the data likely represent early cancers and individuals who were fit to undergo surgery. This limits our interpretation regarding the potential benefits of colonoscopy because we may have missed patients who had a diagnosis of colorectal cancer and whose initial treatment was not surgery, who were not surgical candidates because of comorbidities, or who had surgery performed after the 30-day period. For the latter argument, we think our estimates represent most patients with colorectal cancer who underwent surgical treatment in Ontario, given that according to the Canadian Institute for Health Information the median waiting time for colorectal surgery in Ontario was 17 days in 2016 and that 90% of patients underwent surgery within 34 days.^38^ Finally, we were unable to obtain individual data on type of sedation, endoscopists’ experience and specialty, polypectomy description, and histology. These are important variables that could influence outcomes in patients undergoing colonoscopy. On the other hand, these are not strictly patients’ baseline characteristics and, therefore, are perhaps less relevant to the decision of whether to perform a colonoscopy in an elderly patient for the first time. We think that more detailed information on patients’ comorbidities, long-term medications, and polypectomy procedures, which were not available in the current study, should be considered for inclusion in future research to explain our observations that older patients have a higher incidence of postcolonoscopy bleeding and a higher overall incidence of postcolonoscopy complications.

## Conclusions

In this study, patients aged 75 years and older appeared to have a higher risk of postcolonoscopy complications during the 30-day period after colonoscopy compared with screen-eligible patients. Baseline comorbidities were independently associated with complications, and cardiovascular-related diagnoses were among the most common causes of hospital admission in the older cohort. In accordance with our findings, the decision to perform colonoscopy should be considered carefully in older patients, particularly in the presence of comorbidities.

## Notes

Supplement. **eAppendix.** Supplementary Methods **eFigure.** Probability of 30-Day Complications **eTable 1.** Databases and Coding **eTable 2.** Detailed Description of Cardiovascular-Related Admissions **eTable 3.** Matched Analysis According to Number of Comorbidities and Frequencies of Comorbidities for Older and Younger Patients **eTable 4.** Rates of Surgically Treated Colorectal Cancer According to Type of Colonoscopy
