# Supplementary material for: Association Between Age and Complications After Outpatient Colonoscopy
Source: JAMA Netw Open. 2020 Jun 25;3(6):e208958. doi: 10.1001/jamanetworkopen.2020.8958 (PMC7317606; doi:10.1001/jamanetworkopen.2020.8958)
Supplement: Supplement. — eAppendix. Supplementary Methods eFigure. Probability of 30-Day Complications eTable 1. Databases and Coding eTable 2. Detailed Description of Cardiovascular-Related Admissions eTable 3. Matched Analysis According to Number of Comorbidities and Frequencies of Comorbidities for Older and Younger Patients eTable 4. Rates of Surgically Treated Colorectal Cancer According to Type of Colonoscopy [file jamanetwopen-3-e208958-s001.pdf]

Supplementary Online Content

Causada-Calo N, Bishay K, Albashir S, Al Mazroui A, Armstrong D. Association between age and complications after outpatient colonoscopy. *JAMA Netw Open*. 2020;3(6):e208958. doi:10.1001/jamanetworkopen.2020.8958

eAppendix. Supplementary Methods
eFigure. Probability of 30-Day Complications
eTable 1. Databases and Coding
eTable 2. Detailed Description of Cardiovascular-Related Admissions
eTable 3. Matched Analysis According to Number of Comorbidities and Frequencies of Comorbidities for Older and Younger Patients
eTable 4. Rates of Surgically Treated Colorectal Cancer According to Type of Colonoscopy

This supplementary material has been provided by the authors to give readers additional information about their work.

© 2020 Causada-Calo N et al. *JAMA Network Open.*

eAppendix. Supplementary Methods

The association between older age (>75 years old) and complications after colonoscopy could be confounded by baseline comorbidity. In order to deal with this limitation, we performed a multivariable logistic regression model including individual comorbidities (main analysis and results). In order to assess the potential confounding effect of comorbidity we performed a secondary analysis by matching exposed and non-exposed patients (i.e.: >75 versus < 75) on baseline comorbidity. Initially, we created a numerical variable indicating the number of comorbidities. Second, we matched every patient older than 75 years old with a patient younger than 75 years old with the same number of comorbidities. For example, for every patient above the age of 75 with 0 comorbidities, we selected a patient <75 with 0 comorbidity. For every patient >75 with one comorbidity, we selected a <75 with one comorbidity, and so forth. Once our matched cohort was created, we assessed the association of age and colonoscopy complications using generalized estimating equations (to account for clustering within each match pair).
Out of the 7626 patients older than 75 years of age, 7569 (99.2%) could be matched with a pair younger than 75 years old. **eTable 4** shows the frequency of comorbidities in older and younger than 75 years. As depicted from this table, within each strata of comorbidity, the number of exposed and non-exposed was equal.

© 2020 Causada-Calo N et al. *JAMA Network Open.*

eFigure. Probability of 30-Day Complications(*)

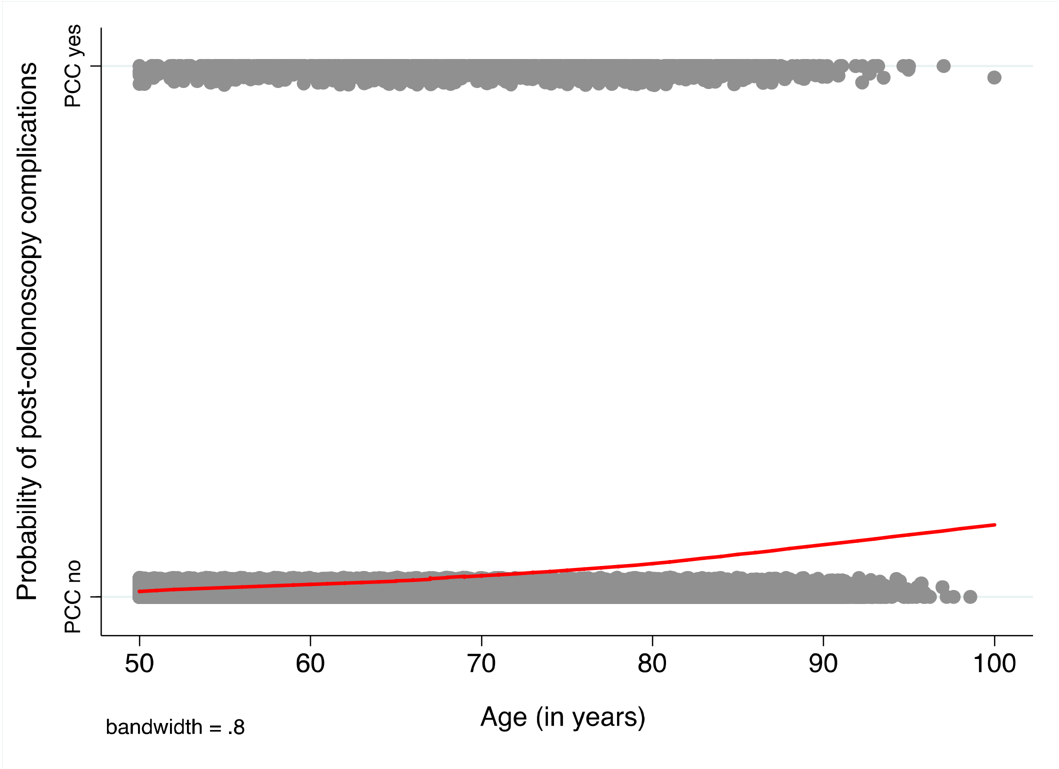

(*) Lowess function (local weighted regression to fit a smooth curve through points in a scatter plot) to explore the relationship between age as a continuous variable, and the predicted probability of post-colonoscopy complications at 30 days.

© 2020 Causada-Calo N et al. *JAMA Network Open.*

eTable 1. Databases and Coding

Term	Database(s)	Codes
Colonoscopy [24]	OHIP; CIHI-DAD and same day surgery	**OHIP**: Z codes (Z555A, Z491A-Z499A), **except** Z555A+/-E740A alone and Z496A+/-E740A alone<br>**CIHI DAD/SDS**: 2NK70, 2NK71, 2NM70, 2NM71, 2NQ70, 2NQ71, 1NK87, 1NM59, 1NM87, 1NQ59,1NQ87, 1NQ89
Surgically -treated colorectal cancer	Ontario Cancer Registry and CIHI-DAD	C18.x (NOT C18.1), C19, C20 **AND** Large bowel excision.
Post-colonoscopy bowel perforation	CIHI	Admission following colonoscopy that included the following:<br>T812 **NOT K631**
Post-colonoscopy bleeding [31]	CIHI	Admission following colonoscopy with any of the following diagnoses:<br>T810, K625, D62, K921, K922, R58 **AND** with any of the following conditions:<br>- Patients with no alternative procedures that could represent the cause of bleeding.<br>- Patients with procedures performed during the hospitalization that would likely be done to treat bleeding (e.g. surgery).
Chronic kidney disease	CIHI	N18
Heart failure	CIHI	I 50 (I 50.0; I 50.1; I 50.9)
Hypertension	CIHI	I10; I15
Atrial fibrillation and flutter	CIHI	I48x
Other cardiac arrhythmias	CIHI	I44; I45; I47; I49
Coronary heart disease	CIHI	I20x; I25x

© 2020 Causada-Calo N et al. *JAMA Network Open.*

Anemia	CIHI	D50; D51; D52; D53
Smoking	CIHI	Z72.0; F17.2, Z87.891
COPD	CIHI	J44x
Obesity	CIHI	E66x
Liver disease	CIHI	K70x, K74.6, K76.0, K75.8, K71; K72; K73; K74; K75; K76; K77
Personal history of malignant neoplasm of the digestive organs	CIHI	Z85.09
Inflammatory bowel disease	CIHI	K50x; K51x; K52x
Polyposis syndrome	CIHI	D12.6; Q85.8

© 2020 Causada-Calo N et al. *JAMA Network Open.*

eTable 2. Detailed Description of Cardiovascular-Related Admissions

CV diagnoses	All CV-related admissions (n=274)	Admissions in the age group 50-74 (n=138)	Admissions in the age group ≥ 75 (n=136)
Heart failure (n, %)	90 (32.8)	38 (27.5)	52 (39.0)
Valvular disease (n, %)	9 (3.3)	3 (2.2)	6 (4.4)
Cardiac arrhythmia (n, %)	29 (10.6)	14 (10.1)	15 (11.0)
Myocardial infarction (n, %)	54 (19.7)	35 (25.4)	19 (14.0)
Thromboembolic disease (n, %)	50 (18.2)	25 (18.1)	25 (18.3)
Acute kidney injury (n, %)	22 (8.0)	14 (10.1)	8 (5.9)
Endocarditis (n, %)	8 (3.0)	6 (4.4)	2 (1.5)
Miscellanea (n, %)	12 (4.4)	5 (2.2)	8 (5.9)

CV: cardiovascular

© 2020 Causada-Calo N et al. *JAMA Network Open.*

eTable 3. Matched Analysis According to Number of Comorbidities and Frequencies of Comorbidities for Older and Younger Patients

**Matched cohort**		
	≥ 75 years old (N=7569)	< 75 years old (N=7569)
0 comorbidities	n=5918	n=5918
1 comorbidity	n=1209	n=1209
2 comorbidities	n=263	n=263
3 comorbidities	n=97	n=97
4 comorbidities	n=49	n=49
5 comorbidities	n=22	n=22
6 comorbidities	n=8	n=8
7 comorbidities	n=3	n=3

© 2020 Causada-Calo N et al. *JAMA Network Open.*

eTable 4. Rates of Surgically Treated Colorectal Cancer According to Type of Colonoscopy

	Index colonoscopy	Surveillance colonoscopy	P-value(*)
**All (N= 38,069)**	219/27,831 (0.8)	44/10,238 (0.4)	< 0.001
**CRC-screen eligible cohort (n= 30,443)**	120/22,566 (0.5)	24/7877 (0.3)	0.01
**Elderly cohort (n= 7,626)**	99/5265 (1.9)	20/2361 (0.9)	0.001

(*) Chi-squared test

© 2020 Causada-Calo N et al. *JAMA Network Open.*
